# Fungal Keratitis in a Critically Ill Post-trauma Patient

**DOI:** 10.7759/cureus.42822

**Published:** 2023-08-01

**Authors:** Sarah L Pulliam, Martha S Nkangabwa, Rebekah Lantz, Asif Khan

**Affiliations:** 1 General Medicine, Wright State University Boonshoft School of Medicine, Dayton, USA; 2 Clinical Research, Wright State University Boonshoft School of Medicine, Dayton, USA; 3 Internal Medicine, Miami Valley Hospital, Dayton, USA; 4 Infectious Disease, Dartmouth Hitchcock Medical Center, Dartmouth, USA

**Keywords:** infectious disease pathology, staph lugdunensis, mva (motor vehicle accident), helmeted injury, trauma, fungal keratopathy, fungal endophthalmitis, candida albicans keratitis, keratitis trauma, fungal keratitis

## Abstract

Keratitis is the leading cause of corneal blindness in the world. Nearly half the cases are due to a fungal infection known as fungal keratitis (FK). There is much variability in the clinical presentation of FK, so diagnosis can be difficult. With the risks of blindness in disease progression being so high, it is vital to diagnose and treat FK quickly. We present a case of FK due to *Candida albicans* and *Staphylococcus lugdunensis-oxa ss* after a motor vehicle accident, its treatment, and the general outcome.

A 71-year-old man with a history of hypertension, hyperlipidemia, arthritis, and previous tobacco use presented after a helmeted motorcycle accident with back pain and bilateral lower extremity sensory and motor function loss. He suffered many fractures and was in neurogenic shock. He had nearly daily reduction and fixation of multiple axial spinal fractures while in the surgical intensive care unit and was ultimately unable to be successfully extubated. Between two intubations, he complained to his family of blurry vision, and there was notable purulence and corneal haziness in bilateral eyes.

The healthcare team initially suspected the eye infection was due to a bacterial etiology, and he was subsequently diagnosed with *Pseudomonas pneumonia* on respiratory cultures. However, several days of antibiotics did not improve the ocular exam. A corneal culture was positive for *C. albicans* and *S. lugdunensis-oxa ss,* and anti-fungal treatment was initiated with ocular improvement. Unfortunately, the patient succumbed to his injuries and further sepsis at another site. With a progressively poor prognosis and machine dependence, he was made do-not-resuscitate per family wishes and died within two hours after cessation of hemodialysis.

One of the greatest barriers to diagnosing FK in the United States is the absence of information regarding the disease. Though novel diagnoses and treatment strategies are in development, the fungal etiology of keratitis should be included in the curricula for not just medical students but also for providers and specialists, as the incidence of FK continues to grow with globalization. We also aim to emphasize the importance of a multidisciplinary team in these cases, as ophthalmology and infectious disease specialists should be involved immediately in order to improve patient outcomes.

## Introduction

Infectious inflammation of the cornea, otherwise known as keratitis, is the leading cause of corneal blindness in the world [1-2]. This type of keratitis is often caused by four types of organisms: bacteria, viruses, protozoa, and fungi, with nearly half of the cases attributed to fungi. The fungi that cause monomorphic fungal keratitis (FK) can often be stratified into filamentous or yeast classes. The most common offenders among the filamentous types globally are *Aspergillus spp.* and *Fusarium spp.*, while *Candida spp*. often dominates in the yeast category [3]. Patients with FK may present with eye pain, eye discharge, eye redness, blurred vision, blepharospasm, hypopyon, and corneal ulcerations. These ulcerations can be characterized by "elevated firm slough, hyphate lines that extend beyond the edge of the central ulcer, and feathery satellite stromal lesions" [4].

Given the variability of presentation and course, diagnosis of FK can be tricky, which is why it is often discovered at later stages of infection [2,5-6]. Fungal keratitis can be diagnosed by microscopic examination, polymerase chain reaction tests, and corneal scraping cultures [3,7]. Fungal keratitis pathophysiology can lead to corneal destruction and blindness. This often comes in the form of corneal perforation, which can usually only be treated with surgery [2,7]. Infection of the vitreous or aqueous humor, also known as endophthalmitis, is a medical emergency associated with FK that can lead to blindness within hours of symptom onset [3]. Even with successful treatment, corneal scarring and opacification in recovery can permanently affect vision [7].

The case presented in this paper is an example of the convoluted path taken to reach a diagnosis of FK. In our patient, *Candida albicans* and *Staph lugdunensis-oxa ss* were observed in corneal scrapings after much investigation, and as a result, their treatment plan was modified.

## Case presentation

The patient, a 71-year-old Caucasian man, had a medical history significant for hypertension, hyperlipidemia, arthritis, and previous tobacco use. His home prescriptions included amlodipine, pantoprazole, triamterene-hydrochlorothiazide, atenolol, potassium chloride, tadalafil, dicyclomine, and aspirin. He was reported to wear readers per family; otherwise, he had no known ocular, autoimmune, or immunocompromised risk factors. The patient presented to the emergency department after a motorcycle accident while wearing a helmet and was notably complaining of back pain and sensory and motor function loss of the bilateral lower extremities. He denied any ocular complaints at this time. Several fractures were noted on imaging, including in the thoracic and cervical vertebra, multiple ribs, right femur and acetabulum, and right first metacarpal bone. He was expedited to the surgical intensive care unit (SICU) for the reduction and internal fixation of his thoracic spinal injuries. During this time, the patient decompensated secondary to hypovolemic shock and was intubated. He was started on cefoxitin for open fracture prophylaxis.

**Figure 1:**
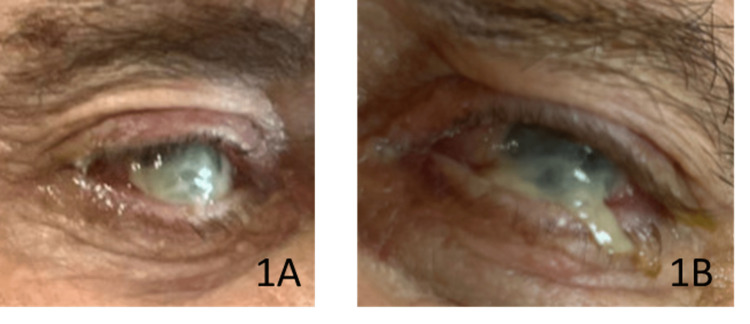
Right eye (OD) (1A) and left eye (OS) (1B) on hospital day five, when ocular symptoms were first documented OD: oculus dexter; OS: oculus sinister

On hospital day three, the patient was stable enough to be extubated. At this time, he complained of blurry vision and was noted to have a purulent discharge of bilateral eyes (oculus uterque (OU)), so bilateral saline was flushed four times daily. On hospital day five, however, the patient again experienced respiratory decline and was re-intubated. His eye exam had not improved, as pictured in Figure 1.

It was on this fifth day of admission that ophthalmology was consulted. The slit lamp exam showed similar results bilaterally: 1+ blepharitis, 2+ injection and chemosis, and significant corneal haze throughout with circular ring opacity. There were no foreign bodies, follicles, or papillae seen. The ophthalmology team suspected a diagnosis of bacterial conjunctivitis, and he was started on moxifloxacin and brimonidine eyedrops. The patient was also started on linezolid and piperacillin/tazobactam with respiratory cultures growing *Pseudomonas pneumonia*.

**Figure 2:**
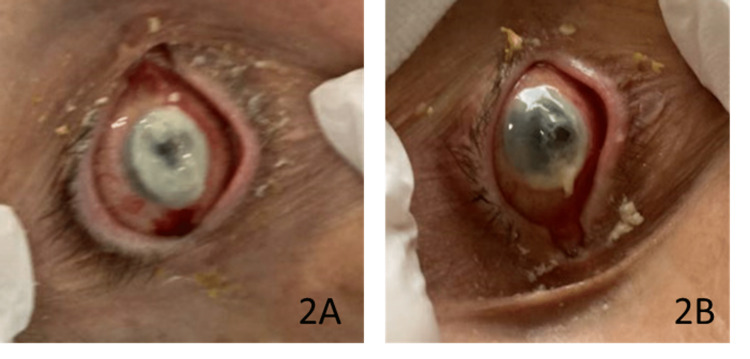
Right eye (OD) (2A) and left eye (OS) (2B) on hospital day seven, at the time of initiating antifungal treatment OD: oculus dexter; OS: oculus sinister

By hospital day six, the multidisciplinary team suspected his ocular findings were related to bacterial keratitis secondary to the *Pseudomonas *infection. Infection was postulated to seed through to the cornea. Thus, moxifloxacin was discontinued, and vancomycin and ceftazidime eyedrops were started. Corneal cultures were also obtained at this time. It was noted on the physical exam that after changing the antibiotic regimen, his corneal ulcers were minimally improving, even after several debridements. The following day, the patient’s eyes were not improving, as pictured in Figure 2.

**Figure 3:**
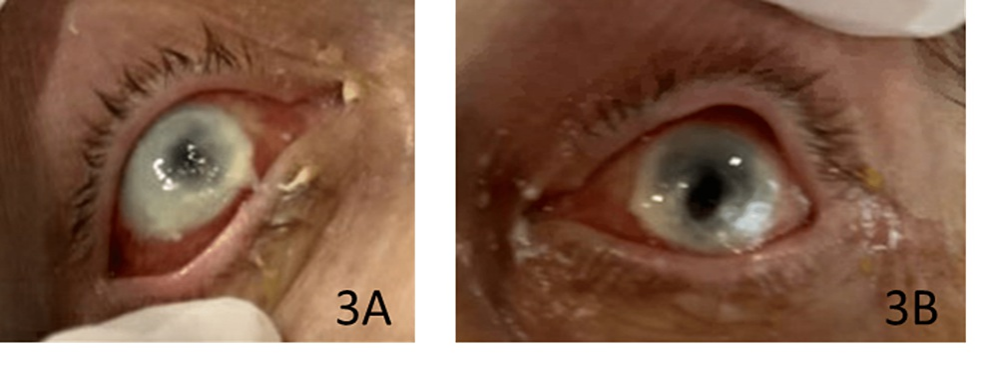
Right eye (OD) (3A) and left eye (OS) (3B) on hospital day eight, the day following antifungal therapy OD: oculus dexter; OS: oculus sinister

The corneal labs came back without significant Pseudomonas growth but were positive for *Candida albicans* in the right eye (oculus dexter (OD)) and *Staphylococcus lugdunensis-oxa ss* in the left eye (oculus sinister (OS)). Upon microscopic examination, it was noted that there was "feet morphology" consistent with *C. albicans keratitis*. Therefore, fungal infection was now at the top of the differential diagnostic considerations after nearly a week of symptoms. Voriconazole 1% ophthalmic solution, one drop every hour, and oral fluconazole 800 mg daily were prescribed with a good response, and haziness improved within one day, as shown in Figure 3.

**Figure 4:**
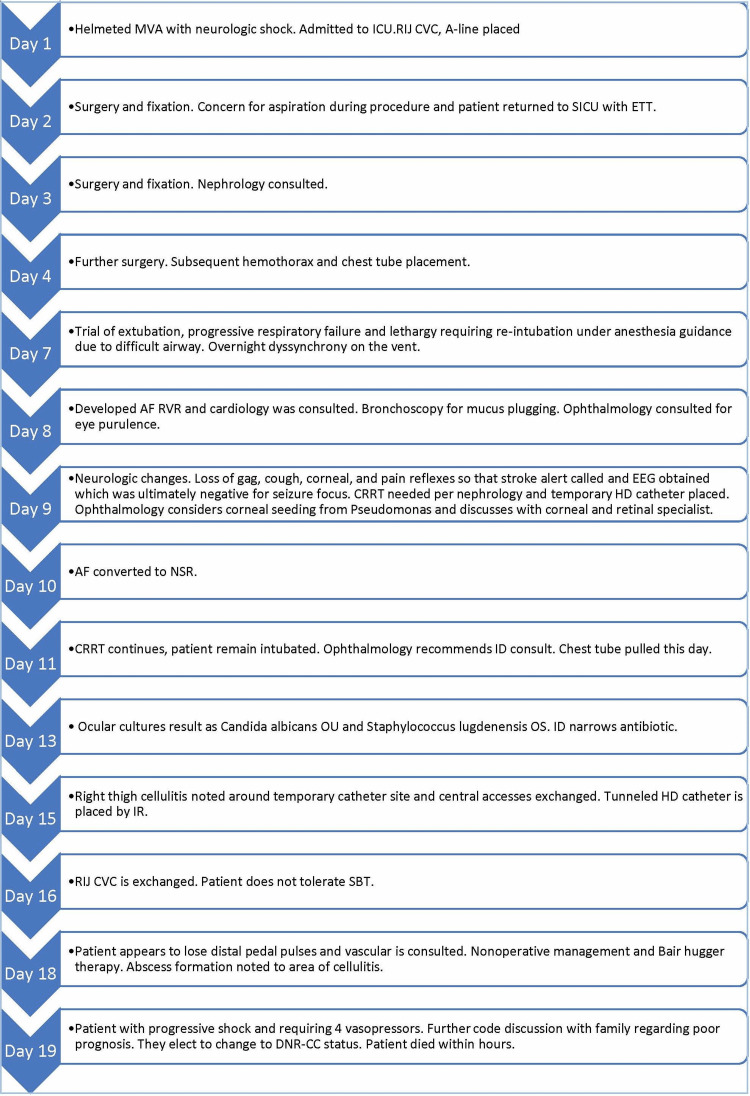
Chronology of the patient’s hospital course AF: atrial fibrillation; A-line: arterial line; CRRT: continuous renal replacement therapy; DNR-CC: do not resuscitate-comfort care; EEG: electroencephalogram; ETT: endotracheal tube; ICU: intensive care unit; SICU: surgical ICU; ID: infectious disease; IR: interventional radiology; MVA: motor vehicle accident; NSR: normal sinus rhythm; OS: oculus sinister (left eye); OU: oculus uterque (both eyes); RIJ CVC: right internal jugular central venous catheter; RVR: rapid ventricular response; HD: hemodialysis; SBT: spontaneous breathing trial

Over the following week, the patient’s eye exam continued to improve, including less inflammation and exudate, and he had improved vision field testing. However, improvement was met with complications, and the patient developed cellulitis in the right thigh around a hemodialysis catheter. Despite the tunneled catheter being exchanged as a potential further infectious source, he also had ICU myopathy, so he could only support short spontaneous breathing trials before tiring. Abscess formation occurred at the site despite empiric treatment; he progressed to septic shock, requiring four vasopressors; and continuous renal replacement therapy had to be discontinued, despite the fact that it was imperative for metabolic acidosis clearance, in order to maintain the patient’s mean arterial pressure. The patient neurologically decompensated and was changed to do-not-resuscitate status with comfort care per family request after detailed discussions regarding his poor prognosis. After the cessation of aggressive resuscitation therapies, the patient passed within hours. The patient's chronology is shown in Figure 4.

## Discussion

One of the biggest hurdles in diagnosing FK is the prevalence of mono-bacterial and bacterial-fungal coinfections. A study in the United Kingdom over the span of a decade revealed that 27% of cases of FK were treated as a mixed bacterial-fungal infection, similar to our patient [2]. However, studies have demonstrated key differences between bacterial and fungal keratitis. Fungal keratitis shows less purulence in inflammation as it often has a lower neutrophil prevalence than bacterial keratosis (BK). Fungal keratitis also tends to have a slower progression than BK [7]. Treatment outcomes also differ between FK and BK. Fungal keratitis has poorer visual outcomes, increased hospitalizations, longer healing times, more complications, more failed treatments, and significantly higher medication costs than BK [2,7-8]. One study showed that the median re-epithelialization of the cornea was 25 days in BK compared to FK at 30 days [9]. Many of these outcome differences can be attributed to the delay seen in diagnosing and treating FK in comparison to BK. Though BK is more common, the number of cases has been declining [10]. On the other hand, FK incidence has increased over the past 30 years, making it important to include FK as a differential diagnosis of eye infection [7,10].

The difficulty of diagnosing and treating FK stems from several issues, including a general lack of awareness and education in infectious disease and ophthalmology training [3-4]. Empiric treatments for bacterial eye infections include corticosteroids and antibiotics, which have little to no effect on FK and increase the risk of progression [7,11]. Treatment proves difficult because it can be hard to penetrate the cornea with medications. Consequently, most treatments for FK are fungistatic rather than fungicidal [1].

Identifying risk factors for FK is essential to its diagnosis and prevention. In the decade-long United Kingdom study, all patients diagnosed with some form of FK had at least one risk factor, and our patient was no exception. A common factor for developing FK in intensive care unit (ICU) patients is an ocular surface disease, principally among patients on ventilation or receiving sedatives or neuromuscular relaxants. Sedatives and relaxants decrease eyelid reflex and can cause incomplete closure of the eyelids, resulting in the eyes drying out faster. Dry eyes are more susceptible to ocular surface diseases. This is especially true for *Candida spp.*, the fungi discovered in our patient’s corneal scrapings [3]. Patients may have other underlying predispositions to dry eyes. Antihistamines, atropine, phenothiazines, and tricyclic antidepressants, often used in the ICU, can decrease tear duct activity. Patients that are unconscious in the ICU additionally cannot express eye complaints, allowing ocular diseases to progress without notice [12]. Our patient also experienced possible corneal trauma with the motor vehicle accident, giving another risk for an FK exposure [7]. Although not present in our case, additional risk factors include socioeconomic status with a higher rate of complications, likely due to delays in healthcare and increased exposure [7-8], and injection drug use due to a lower threshold for systemic fungal disease [3].

We believe, in our case, that the patient’s FK was identified early enough to prevent surgical intervention. In a less critically ill patient, he may have preserved his vision and survived without the other comorbid injuries and concomitant infections. In cases where more than a third of the posterior cornea is involved, the risk of corneal perfusion with hypopyon significantly increases [2]. In addition, if the patient contracts endophthalmitis, there is generally no good prognosis for full recovery [7]. Given the high medical treatment failure rate for FK, it often portends a poor prognosis [5]. Additional prognostic contributors are age and the initial size of the infiltrate. One study found significantly worse vision outcomes after three months of treatment in those who were elderly and had a larger infiltrate [2].

As the prevalence of FK rises globally, novel diagnosis and treatment techniques are evolving. Traditionally, molecular and microscopic techniques are used to identify the fungus, with molecular techniques being more reliable [4]. Presently, the polymerase chain reaction test is the most accurate diagnosis method, but it is often expensive and may not be accessible in less-populated areas [3]. Restriction fragment length polymorphisms and next-generation sequencing to identify the fungi species are being investigated [3,6]. Existing non-surgical interventions are limited for those with severe disease and have a high failure rate [6]. Antimicrobial peptide, collagen cross-linking, and phage therapies seem promising as noninvasive treatments for FK [2,10].

Healthcare protocols have evolved to help reduce time in the diagnosis of FK. In one case study, there was a post-intravitreal injection outbreak of FK due to preparations at a compound pharmacy. However, changing protocols and having stricter regulations helped raise awareness and reduce the incidence of FK [3]. Another study examined ICU patients and implementing protocols to promote eye health during their stay in the hospital. Patients in the ICU, especially those who are comatose or are on ventilation, are at much higher risk than the general population for FK. The protocols in this study were used to reduce the risk of ocular surface disease. Methods included taping patients’ eyes shut, using ophthalmic lubricants such as 0.3% hypromellose teardrops, and covering the eyes with contact lenses and polyethylene rings. Through these practical and low-maintenance protocols, the incidence of FK and other ocular diseases in the ICU decreased dramatically [12].

## Conclusions

Once thought to be a disease exclusive to the tropics, FK is a growing issue as our world continues to globalize. Given the high medical treatment failure rate and continued incidence, it is important that this condition be suspected and detected early. A common reason for the delay in FK diagnosis is a lack of provider awareness. Consequently, it is imperative that FK be incorporated into infectious disease and ophthalmology training courses. Protocols including ICU eyecare and continuing to discuss fungi in differentials of eye infections have proven useful in early disease detection and more successful treatment outcomes. We hope to draw awareness to this uncommon ocular fungal infection in order to encourage novel diagnostic methods and treatments. Early treatment means less transfer of infectious diseases, and the goal would be to reduce and eliminate cases in the United States and globally.
